# Histological regression of peritoneal metastases of recurrent tubo-ovarian cancer after systemic chemotherapy

**DOI:** 10.3389/fsurg.2022.936613

**Published:** 2022-09-23

**Authors:** Basile Pache, Hugo Teixeira Farinha, Laura Toussaint, Nicolas Demartines, Delfyne Hastir, Patrice Mathevet, Christine Sempoux, Martin Hübner

**Affiliations:** ^1^ Department of Visceral Surgery, Lausanne University Hospital (CHUV), University of Lausanne (UNIL), Lausanne, Switzerland; ^2^ Gynecology Unit, Department Women-Mother-Child, Lausanne University Hospital (CHUV), University of Lausanne (UNIL), Lausanne, Switzerland; ^3^ Faculté de Biologie et Médecine, University of Lausanne, Lausanne (UNIL), Switzerland; ^4^ Department of Pathology, Lausanne University Hospital (CHUV), University of Lausanne (UNIL), Lausanne, Switzerland

**Keywords:** surgery, PRGS, peritoneal metastasis, chemotherapy, gynecology, ovary, histology

## Abstract

**Introduction:**

Post-treatment histological regression of peritoneal metastases (PM) is a new and potentially important predictor of oncological outcomes. Histology of PM from adnexal origin is usually evaluated by the Chemotherapy Response Score (CRS). The aim of this preliminary study was to quantify the response of PM of recurrent tubo-ovarian cancer (TOVC) after systemic chemotherapy by using the recently validated Peritoneal Regression Grading System (*PRGS*) and compare it with CRS. Correlation with per operative evaluation through Peritoneal Cancer Index (PCI) was performed.

**Material and methods:**

Retrospective cohort study of all consecutive patients with recurrent PM from TOVC undergoing surgery after prior systemic chemotherapy from January 2015 to March 2019. Biopsies were assessed with the four-scale PRGS.

**Results:**

Thirty-eight patients were included. Patients had a median of 2 (range 1–2) lines and 12 (range 3–18) cycles of prior systemic chemotherapy. Overall mean (SD) PRGS was 2.3 (±1.1). Of the patients, 26% (10) had complete response (PRGS 1), 40% (15) had major response (PRGS 2), 26% (10) minor response (PRGS 3), and 8% (3) had no response (PRGS 4). Mean PRGS was positively correlated with the Peritoneal Cancer Index (*ρ* = 0.5302, *p* = 0.0003) and inversely correlated with CRS (*ρ* = −0.8403, *p* < 0.0001). No correlation was highlighted between mean PRGS and overall survival (*ρ* = −0.0195, *p* = 0.9073).

**Conclusion:**

CRS and mean PRGS correlated with each other. Histological response of PM after systemic chemotherapy was quantifiable and variable. The role of PRGS for the evaluation of treatment response and as potential surrogate marker for oncological outcomes is part of ongoing and planned research.

## Introduction

Peritoneal metastases (PM) are present in up to 60% of all gynecologic tumors at the time of diagnosis (1, 2). Life expectancy of patients with PM is limited and depends mainly on the disease extent and response to therapy (3). Resistance of PM to systemic chemotherapy can be explained by molecular mechanisms and by limited drug entry into peritoneal nodules (4).

Evaluation of treatment response remains challenging, as many patients have no target lesions allowing evaluation according to the RECIST criteria (5, 6). Assessment of the response of PM to systemic treatment depends further on the pattern of dissemination and the experience of the radiologist (7). Pressurized intraperitoneal aerosol chemotherapy (PIPAC) is a treatment modality offering simultaneous access to tumor biopsies in patients who, in majority, received systemic chemotherapy prior to surgery (8–10). Tumor spread within the abdominal cavity is surgically assessed using the peritoneal cancer index (PCI) (11). Various scores exist for the histological assessment of PM, mostly specific for one tumor or another (12). It relies usually on characteristics such as fibrosis, acellular mucin pools, hyalinosis, and/or infarct-like necrosis. Specifically for PM from tubo-ovarian cancer (TOVC), the Chemotherapy Response Score (CRS) score was designed in 2015 for tubo-ovarian high-grade serous carcinoma (HGSC), with a three-tier chemotherapy response score depending on the histological response on omental examination (13).

For more reproducibility among the different types of cancers with PM, a novel score was developed in order to assess treatment response of PM on the histological level: the *Peritoneal Regression Grading System* (PRGS) (14, 15). Little is known on histological regression of PM after systemic chemotherapies, and the clinical value of this novel tool remains unclear.

The aim of this preliminary study was to quantify, in patients with peritoneal carcinomatosis of TOVC, histological response of PM after systemic chemotherapy by using the *PRGS score*, compare it with the established CRS and to study potential correlations with clinical variables.

## Materials and methods

We followed the methods of Toussaint et al. (16) and briefly described it hereafter. It is a retrospective cohort study including all consecutive patients admitted for PIPAC after systemic chemotherapy for peritoneal metastasis of TOVC from January to March 2019. Criteria of exclusion were prior PIPAC therapy, no prior systemic chemotherapy, and patients’ refusal to participate. Criteria for PIPAC treatment was discussed during a tumor board, mainly after at least one prior line of systemic chemotherapies, with recurrent/progressive peritoneal carcinomatosis.

### Data management

Data were extracted from a prospectively maintained institutional database and used similar variables than Toussaint et al.

### Biopsy sampling

Biopsies were taken during the PIPAC procedure, before applying intraperitoneal chemotherapy (8, 14).

### Histological response

Systematic use of PRGS was initiated in our institution immediately after publication of the proposal in June 2016 and was used since then on a routine basis. PRGS and CRS were retrospectively assessed for the specimens received before June 2016. As described by Solass et al., biopsies are taken from areas macroscopically suspected for tumor in four abdominal quadrants and the omentum, if technically possible. Biopsies are fixed in 10% buffered formalin and then embedded in paraffin, sliced, and then stained with hematoxylin and eosin. Histological features of regression are fibrosis, inflammation, hyalinosis, acellular mucin pools, ischemic necrosis, accumulation of macrophages/multinucleated giant cells, and granuloma formation. The PRGS score is defined as follows: a score of 1 corresponds to a complete regression with absence of tumor cells; a score of 2 to major regression features with only a few residual tumor cells; a score of 3 to minor regression with predominance of residual tumor cells and only few regressive features; and a score of 4 corresponds to an absence of response to therapy and where the tumor cells are not accompanied by any regressive features. A PRGS score was assessed for each biopsy, as well as a CRS score. The mean PRGS (out of a minimum of four biopsies) was calculated according to current recommendations in order to depict overall histological response (14, 15). According to the most recent publication, PRGS was presented as mean ± SD for patients having three biopsies at least, and with worst PRGS. In addition to that, the highest and lowest grading were reported. To assess if PRGS had an impact on prognosis, PRGS was also plotted against overall survival (OS) defined by peritoneal cancer diagnosis last follow-up and death. The CRS score was designed in 2015 specifically for tubo-ovarian HGSC, on omental biopsies, with a three-tier chemotherapy response score. Patients are allocated in three groups depending on the histological response on omental examination (CRS1: minimal/no response, CRS2: partial response, CRS3: complete/near complete response). Of note, the CRS was initially validated in patients with primary platinum-based neoadjuvant chemotherapy and interval debulking surgery for HGSC (13). Both PRGS and CRS scores were assessed by the same pathology team.

### Statistics and analysis

Categorical variables were reported as frequencies (%) and compared with a Chi-square test. Mann–Whitney *U* test or Student’s *t*-test were used for continuous variables, depending on the distribution type and variance homogeneity. Statistical correlations were tested by use of Pearson's rank correlation. Continuous variables were presented as mean with SD or median with range or interquartile range (IQR) for skewed data. area under the receiver operating characteristic curve curve and Youden index were used to determine thresholds for number of lines and cycles of chemotherapies. All statistical tests were two-sided. *P*-value <0.05 was considered statistically significant. Analyses and graphisms were performed with SPSS v20 (IBM, Armonk, New York, USA) and GraphPad Prism 7.0 (GraphPad Software, Inc., La Jolla, CA, USA).

### Ethical approval

The present study was approved by the institutional review board (CER-VD 2019-00747).

## Results

Thirty-eight consecutive patients having PIPAC were analyzed. Overall median age (IQR) was 65 years (58–70); 28 patients (74%) had an American Association of Anesthesiologists physical status classification system (ASA) score of 2 and 10 (26%) had an ASA score of 3. From 38 patients with tubo-ovarian cancer, there were 23 HGSC (61%), Five low-grade serous carcinoma (13%), five mucinous carcinoma (MC) (13%), four clear cell carcinoma (CCC) (11%), and one endometrioid carcinoma (EC) (2%) cases. Median follow-up (IQR) after surgery (first PIPAC) was 21 months (13.4–26.9) and nine patients (24%) died within this period. Patients had a median of 2 (range 1–2) lines and 12 (range 3–18) cycles of prior systemic chemotherapy treatments. Previous chemotherapy treatments are summarized in Supplementary Table S1.

### Histological response to systemic chemotherapy

Analysis *per biopsy*: A median of four biopsies (range: 3–7) were taken with a total of 169 analyzed specimens. Thirty-one of those (18%) showed no histological regression (PRGS4), while PRGS 3, 2, and 1 (complete regression) were diagnosed in 33 (20%), 31 (18%), and 74 (44%) specimens, respectively. Discrepant findings for the different specimens of the same patient were present in 23 out of 38 patients (61%) (Supplementar Figure S1).

**Figure 1:**
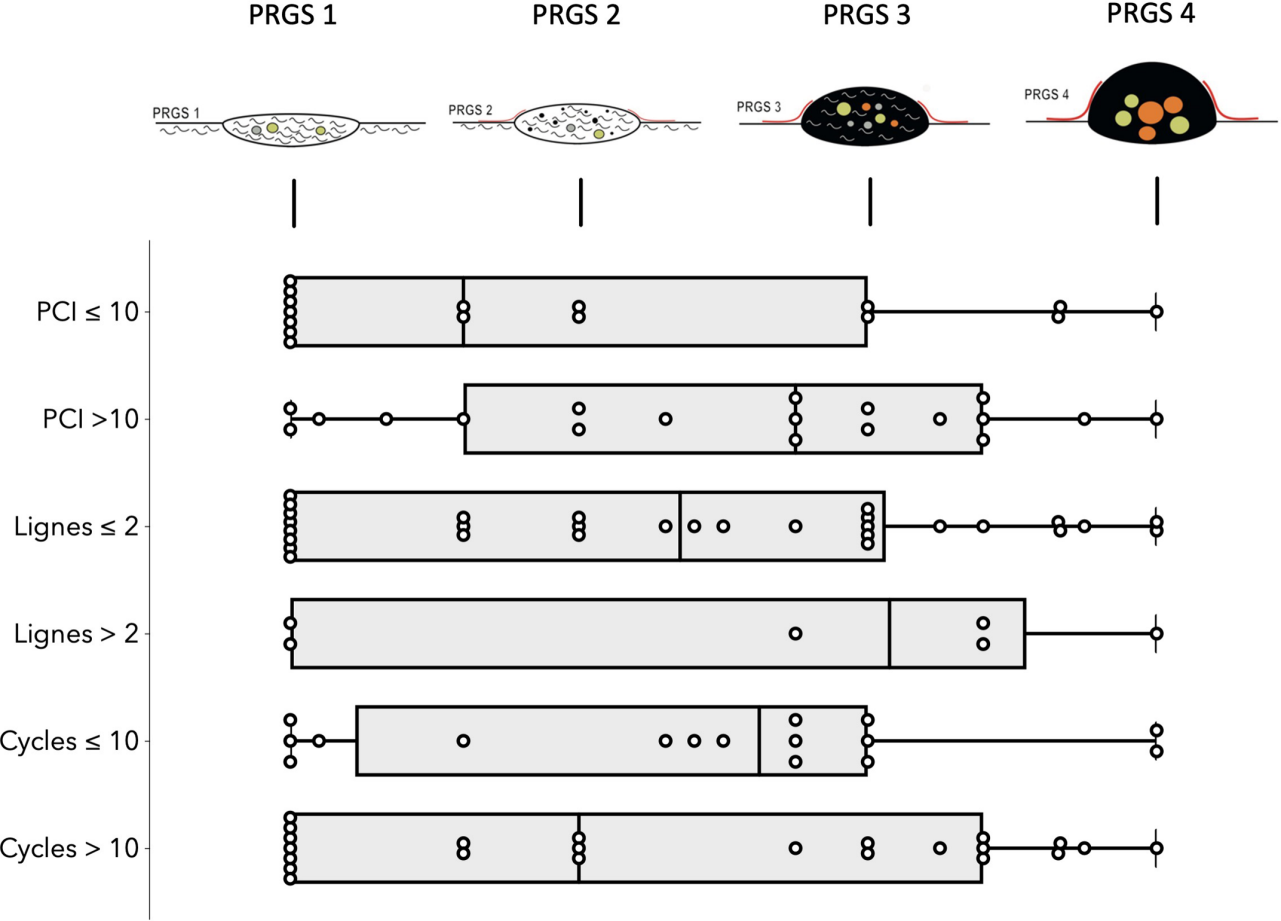
Sensitivity analysis of histological regression (PRGS) of peritoneal cancer after systemic chemotherapy. Horizontal box plots with illustration of highest, lowest, medians, and outliers Peritoneal Regression Grading System (PRGS) response stratified by PCI, lines and cycles of chemotherapies. PRGS-1 corresponds to a complete regression with absence of tumor cells; PRGS-2 to major regression features with only a few residual tumor cells; PRGS-3 to minor regression with predominance of residual tumor cells and only few regressive features; PRGS-4 to no response. PRGS: median, 10th, and 90th percentiles with outlier's data.

Analysis *per patients*: Overall mean (SD) PRGS was 2.4 (±1.1) for the entire cohort. Complete response (PRGS 1) was noted in 10 patients (26%) and no response (PRGS 4) was documented in 3 patients (8%). Major response (PRGS 2) was documented in 15 patients (40%) and minor response (PRGS 3) in 10 (26%). Sensitivity analysis of histological regression (PRGS) by PCI, number of lines, and cycles of chemotherapies are displayed in Figure 1. PRGS was similar in patients having one or two lines of chemotherapy vs. more than two lines before intraperitoneal treatment [mean 2.3 (SD 1.0) vs. 2.6 (SD 1.3), *p* = 0.578]. The same observation was made for patient with 10 or less cycles of chemotherapy compared with more than 10 [mean 2.4 (SD 1.0) vs. 2.3 (SD 1.1), *p* = 0.114]. Seven patients (18%) had last chemotherapy between 2 and 6 weeks before surgery, and 31 (82%) after more than 6 weeks. No difference of PRGS was observed between these two groups [mean 2.5 (SD 0.9) vs. 2.2 (SD 1.2), *p* = 0.511].

**Figure 2:**
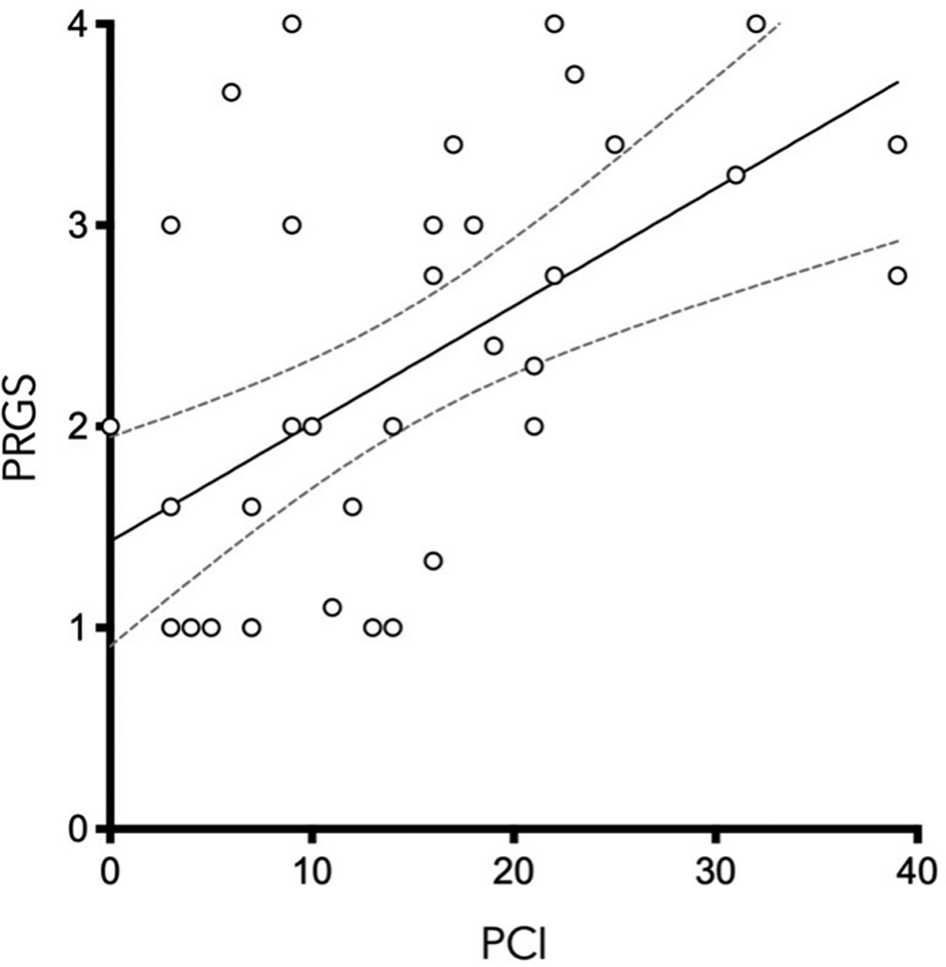
*Peritoneal Regression Grading System* (PRGS) was plotted against the extent of peritoneal disease [measured by the Peritoneal Cancer Index (PCI)] without regard to systemic chemotherapy regimen. *ρ *=* *0.5583, *p *= 0.0003.

**Figure 3:**
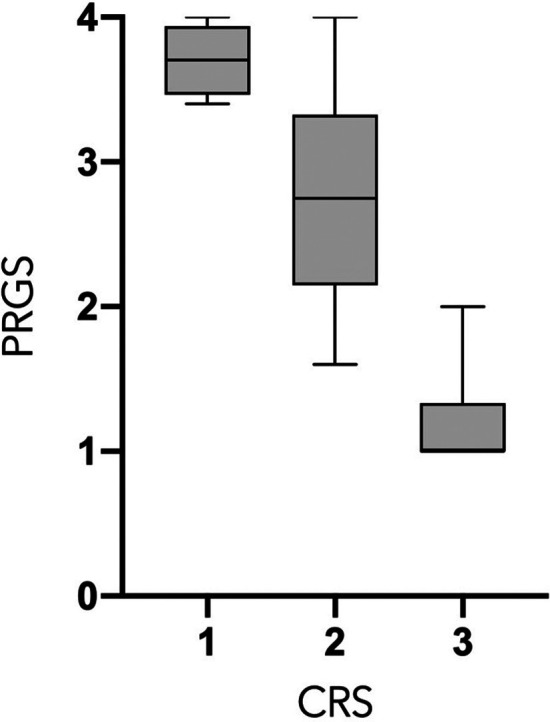
*Peritoneal Regression Grading System* (PRGS) and Chemotherapy Response Score (CRS) correlation. *ρ *=* *−0.8403, *p* < 0.0001.

PCI and mean PRGS were strongly correlated with each other (*ρ* = 0.5302, *p* = 0. 0003), showing an association between advanced disease extent and poor histological regression (Figure 2). In the same way, CRS and mean PRGS correlated with each other (*ρ* = −0.8403, *p* < 0.0001), showing a good association between the two histopathologic scores (Figure 3). CRS was also correlated with PCI (*ρ* = −0.3801, *p* < 0.022).

No relationship was found between PRGS and survival. Median overall survival was 20.4 months (IQR 12.0–22.8). There was no difference between mean PRGS (SD) between deceased patients [2.6 (±1)] and survivors [2.2 (±1.1)] during follow-up (*p* = 0.378). No significant correlation was found plotting mean PRGS against overall survival (months) (*ρ* = −0.0195, *p* = 0.9073).

## Discussion

Moderate histological regression (assessed by PRGS) after systemic chemotherapy was found for this cohort of patients with gynecological malignancies. PRGS was found to be associated with disease extent, but not with prior treatments or survival.

Assessment of treatment response remains a challenge for peritoneal metastasis. Radiological assessment by use of RECIST criteria requires special expertise and does not apply for patients without target lesions. Assessment of histological treatment response might be an interesting surrogate, but until recently, there was neither easy access to representative biopsies nor a validated grading system.

A unified approach to the diagnosis with the PRGS score aims to overcome heterogeneity of scoring malignancies from histological point of view (11, 17, 18). Interestingly, when analyzing different samples for the same patient, heterogeneity in response through PRGS was noted in 60% of patients. It does highlight the importance of multiple biopsies. Discussion can be initiated regarding whether considering the worst PRGS only or to consider the mean PRGS. For futures studies and reproducibility, it is actually advisable to report both (14, 15).

PCI and mean PRGS were strongly correlated (Figure 2), showing an association between advanced disease extent and poor histological regression. Other pathological scores than PRGS have previously been developed for gynecological cancers. Those are based on different methods, such as the three-tier Chemotherapy Response Score (CRS), analyzing fibroinflammatory changes and associated tumor regression for TOVC (13, 19). The comparison of the two scores made in the present study shows a good correlation between them. We can also note that there is a trend in both scores for correlation with PCI, for PRGS (*ρ* = 0.5302, *p* = 0.0005) and for CRS (*ρ* = −0.391, *p* < 0.0152) (Figures 2, 3).

TOVC has a particularly poor prognosis (20, 21). There is actually a debate in its therapeutic interventions. Systemic chemotherapy is the first-line treatment in most cases, with a significant proportion of women who are with little clinical benefits (22), until resistance to treatment is diagnosed (23, 24). Cytoreductive surgery for peritoneal metastasis is then proposed (25). A recent phase 3 trial brought out that after regular carboplatin and paclitaxel systemic treatment of TOVC, the interval cytoreductive surgery could be performed with adjunction of intraperitoneal chemotherapy, in order to maximize drug delivery to peritoneal metastasis (26).

One can imagine that PRGS could be useful for developing new markers of histological response to systemic chemotherapies, or PRGS could—*per se*—predict treatment response of systemic chemotherapies or be used as proxy for survival. Unfortunately, the power of the present study does not allow such development. The correlation of PRGS to clinical characteristics such as clinical stage, platinum-sensitive status, radiological images, or biological tumor markers is of great interest and should be considered for study in larger cohort studies.

Prognostic role of the PRGS remains unstudied, and a large-scale international collaborative study (“PIPAC cohort study”) focuses currently on correlating PRGS scores to the predictive progression-free survival or OS, just as it has been studied with the CRS (Chemotherapy Response Score) (15, 27).

When comparing present results to the literature, gynecological studies focused on PRGS during first biopsies of systemically pretreated patients is to our knowledge nonexistent. Perhaps the PIPAC-OV3 multicenter randomized, phase III trial will give data to compare with the present study. This study, intended as a preliminary study, has several limitations beyond its retrospective nature. First, sample size was small and risk for type II error is relatively high. Then, there was important heterogeneity in terms of demographics and prior treatments. The results of this study have, therefore, to be interpreted with caution. It remains to be awaited if the large-scale PIPAC cohort study will show different Kaplan–Meier survival curves by PRGS grade. Both PRGS and CRS scores were assessed by the same pathology team, but no inter- and intraobserver variability was assessed in the current study, for practical and funding reasons. This could be an interesting prospective project in the future. Limited number of deaths, which is positive, on the one hand, did not allow a dedicated analysis. The most interesting analysis that could be performed would be on comparing PRGS to the categories of patients regarding the indication for surgery. Depending on whether the patient is on relapse, palliative care, or one free interval between two chemotherapies results could be different, opening here an interesting field of research. Finally, PRGS is well defined and easily reproducible (14, 15) allowing future studies to evaluate the prognostic and predictive role of the PRGS, within each subtype of gynecological malignancies.

## Conclusions

CRS and mean PRGS correlated with each other, showing a good association between the two histopathologic scores. The present study confirms that the PRGS is a useful tool to quantify histological regression of PM after prior systemic chemotherapy. Histological regression varied widely, correlated with disease extent but not with prior treatment or survival. Large-scale studies need to clarify the prognostic and predictive potential and hence the clinical value of PRGS in patients with gynecological malignancies.

## Precis

Histological assessment of peritoneal metastasis is challenging. PRGS score offers intuitive evaluation of the latter. PRGS assessment of ovarian PM is feasible.

## Data Availability

The raw data supporting the conclusions of this article will be made available by the authors, upon reasonable request.
