# Erratum: Histological regression of peritoneal metastases of recurrent tuboovarian cancer after systemic chemotherapy

**DOI:** 10.3389/fsurg.2023.1175506

**Published:** 2023-03-09

**Authors:** Frontiers Production Office

**Affiliations:** Frontiers Media SA, Lausanne, Switzerland

**Keywords:** surgery, PRGS, peritoneal metastasis, chemotherapy, gynecology, ovary, histology

An Erratum on Histological regression of peritoneal metastases of recurrent tubo-ovarian cancer after systemic chemotherapy By Pache B, Teixeira Farinha H, Toussaint L, Demartines N, Hastir D, Mathevet P, Sempoux C and Hübner M. (2022) Histological regression of peritoneal metastases of recurrent tubo-ovarian cancer after systemic chemotherapy. Front. Surg. 9:936613. doi: 10.3389/fsurg.2022.936613

An omission to the funding section of the original article was made in error. The following sentence has been added: “Open access funding was provided by the University of Lausanne”.

The original version of this article has been updated.

