# Supplementary material for: Histological regression of peritoneal metastases of recurrent tubo-ovarian cancer after systemic chemotherapy
Source: Front Surg. 2022 Sep 23;9:936613. doi: 10.3389/fsurg.2022.936613 (PMC9632969; doi:10.3389/fsurg.2022.936613)
Supplement: Supplementary file 1 [file Datasheet1.docx]

**Supplementary figure** PRGS response stratified by patient with specimens with discrepant findings


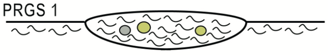

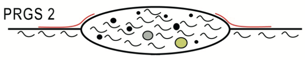

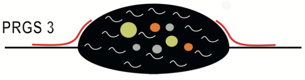

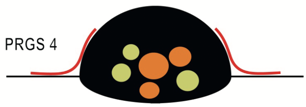


Horizontal box plots with illustration of highest, lowest, medians and outliers Peritoneal Regression Grading System (PRGS) response stratified by patient with specimens with discrepant findings. PRGS-1 corresponds to a complete regression with absence of tumor cells; PRGS-2 to major regression features with only a few residual tumor cells; PRGS-3 to minor regression with predominance of residual tumor cells and only few regressive features; PRGS-4 to no response.

PRGS: median, 10 and 90 percentiles with outlier’s data.
