# Supplementary material for: Histological regression of peritoneal metastases of recurrent tubo-ovarian cancer after systemic chemotherapy
Source: Front Surg. 2022 Sep 23;9:936613. doi: 10.3389/fsurg.2022.936613 (PMC9632969; doi:10.3389/fsurg.2022.936613)
Supplement: Supplementary file 2 [file Table1.docx]

**Supplementary table** Regimen used for the last line of pre-operative chemotherapy

|  | Number of  Patients | Number of  Cycles:  median (range) |
| --- | --- | --- |
| Carboplatin + PTX^1^ | 10 | 6 (3-14) |
| Carboplatin + PTX + Bevacizumab | 7 | 12 (6-18) |
| Carboplatin + PTX + Doxorubicin + Bevacizumab | 6 | 14 (9-18) |
| Carboplatin + PTX + Gemcitabine + Bevacizumab | 5 | 18 (10-18) |
| Carboplatin + PTX + Doxorubicin | 3 | 16 (12-18) |
| Carboplatin + PTX+ Gemcitabine + Doxorubicin + Bevacizumab | 3 | 16 (14-18) |
| Carboplatin + PTX+ Gemcitabine + Oxaliplatin | 2 | 18 (18-18) |
| Carboplatin + PTX + Gemcitabine | 2 | 14 (10-18) |

^1^ PTX: Paclitaxel
